# Supplementary material for: Transient inflammatory response mediated by interleukin-1β is required for proper regeneration in zebrafish fin fold
Source: eLife. 2017 Feb 23;6:e22716. doi: 10.7554/eLife.22716 (PMC5360449; doi:10.7554/eLife.22716)
Supplement: Figure 1—source data 1. — The table shows the numbers of larvae used for evaluating the il1b expression levels in WT or clo mutant at respective time points after fin fold amputation. DOI: http://dx.doi.org/10.7554/eLife.22716.004 [file elife-22716-fig1-data1.docx]

**SOURCE DATA**

**Figure 1** – **Source Data 1. *il1b* expression at respective time points after fin fold amputation.**

| **(n)** | **3 hpa** | **6 hpa** | **12 hpa** | **24 hpa** |
| --- | --- | --- | --- | --- |
| **WT** | **26** | **49** | **24** | **16** |
| ***clo*** | **21** | **52** | **26** | **13** |

The table shows the numbers of larvae used for evaluating the *il1b* expression levels in WT or *clo* mutant at respective time points after fin fold amputation.
