# Supplementary material for: Transient inflammatory response mediated by interleukin-1β is required for proper regeneration in zebrafish fin fold
Source: eLife. 2017 Feb 23;6:e22716. doi: 10.7554/eLife.22716 (PMC5360449; doi:10.7554/eLife.22716)
Supplement: Figure 5—source data 1. — The table shows the numbers of larvae used for evaluating the il1b expression levels in larvae that were injected with respective MOs. DOI: http://dx.doi.org/10.7554/eLife.22716.013 [file elife-22716-fig5-data1.docx]

**SOURCE DATA**

**Figure 5** – **Source Data 1. *il1b* expression in larvae injected with std, *spi1b*, *csf3r*, or *irf8* MOs.**

| **(n)** | **std MO** | ***spi1b* MO** | ***csf3r* MO1** | ***irf8* MO** |
| --- | --- | --- | --- | --- |
| **3 hpa** | **16** | **15** | **18** | **18** |
| **6 hpa** | **26** | **20** | **23** | **19** |

The table shows the numbers of larvae used for evaluating the *il1b* expression levels in larvae that were injected with respective MOs.
